# Hypothesizing the body's genius to trigger and self-organize its healing: 25 years using a standardized neurophysics therapy

**DOI:** 10.3389/fphys.2013.00334

**Published:** 2013-11-19

**Authors:** Sara N. Ross, Ken Ware

**Affiliations:** ^1^Chair of Interdisciplinary Graduate Studies, Antioch University MidwestYellow Springs, OH, USA; ^2^Neurotricional Sciences Pty. Ltd.Gold Coast, QLD, Australia

**Keywords:** fractal physiology, clinical therapy, nervous system, self-reorganization, system transition dynamics

## Abstract

We aim for this contribution to operate bi-directionally, both as a “bedside to bench” reverse-translational fractal physiological hypothesis and as a methodological innovation to inform clinical practice. In 25 years using gym equipment therapeutically in non-research settings, the standardized therapy is consistently observed to trigger universal responses of micro to macro waves of system transition dynamics in the human nervous system. These are associated with observably desirable impacts on disorders, injuries, diseases, and athletic performance. Requisite conditions are therapeutic coaching, erect posture, extremely slow movements in mild resistance exercises, and executive control over arousal and attention. To motivate research into the physiological improvements and in validation studies, we integrate from across disciplines to hypothesize explanations for the relationships among the methods, the system dynamics, and evident results. Key hypotheses include: (1) Correctly-directed system efforts may reverse a system's heretofore misdirected efforts, restoring healthier neurophysiology. (2) The enhanced information processing accompanying good posture is an essential initial condition. (3) Behaviors accompanying exercises performed with few degrees of freedom amplify information processing, triggering destabilization and transition dynamics. (4) Executive control over arousal and attention is essential to release system constraints, amplifying and complexifying information. (5) The dynamics create necessary and in many cases evidently sufficient conditions for the body to resolve or improve its own conditions within often short time periods. Literature indicates how the human system possesses material self-awareness. A broad explanation for the nature and effects of the therapy appears rooted in the cascading recursions of the systems' dynamics, which appear to trigger health-fostering self-reorganizing processes when this therapy provides catalytic initial conditions.

## Introduction

This article's contribution operates bi-directionally, both as a “bedside to bench” reverse-translational physiological hypothesis and as a methodological innovation to inform clinical practice. Mature with 25 years of clinical use without research settings, formal studies are needed to account for why it works, to explicate physiological changes, and to validate the effects of the physical therapy called Ware K Health Trigger Process. Since we find no similar therapies or human body dynamics of the kind reported in the literature, we seek to motivate such research interest by hypothesizing scientific explanations for the therapy's relationship with the body's system dynamics and evident health effects.

The standardized, non-manipulative therapy pairs conventional gym equipment with unconventional techniques that release habitual restraints in the body. The body's response to the properly performed techniques is a series of observable self-organizing transitions at micro and macro levels described below. These phenomena appear to catalyze (if not also represent) significant healing processes in clients with a wide range of debilitating conditions, injuries, and diseases. The same processes also improve athletic performance. When afforded the health-triggering conditions and supported by the therapy's maintenance program, observations and client self-reports indicate numerous cases where the human system can evidently resolve many of its own issues without medication, surgery, or limb manipulation.

To set the contexts of our hypothesizing, the article begins with the therapy's historical background, an introduction to observed system dynamics triggered by the method, and a two-part review section. The first part reviews major issues in relevant fields, from which we conclude that ready explanations for our topic cannot yet exist. On that basis, we next give the major theoretical orientations on which our effort rests. The second half of the article is dedicated to the hypothesis building. After describing the therapy's method and key premises, the remainder presents our literature-based hypothesized explanations, suggestions for ways to test a number of them, and a concluding synthesis of conclusions about the body's genius.

## Background

Ware discovered the trigger for the system dynamics and some of their effects in 1982 during personal body building efforts, and experimented alone until 1988. He continued to explore the phenomena while working at a Queensland hospital's rehabilitation center for physical and emotional disorders and at his own gym facility thereafter. Through word of mouth, many approached Ware as a last resort when conventional treatments failed or were feared (e.g., surgery). While we realize the limitations of referring to cases that occurred outside of formal clinical studies, the clinical track record is that the therapy appears to have observably benefitted the functional status of thousands of individuals, young and old, in connection with dozens of different health problems (Table 1), including athletes realizing significant sports performance enhancement not achieved via their traditional sports exercise approaches. For example, the approach restored functionality to broken legs with poor prior recovery, resulting in world championships in powerlifting (http://nprsr.qld.gov.au/get-active/pdf/women-girls/katrina-robertson.pdf). Among recent cases were a functional recovery from surgical removal of the sciatic nerve and many sensory and motor neuron projections, and restoration of sensation and function in a long-term T12 partial paraplegic (see http://vimeo.com/cloudvision/review/77355465/cb5a2062fe). These and others are viewable at http://f1000.com/posters/browse/summary/1093372.

**Table 1:** **Client conditions evidently benefitted by the therapy**.

Arthritis	Muscular dystrophy
Bipolar disorder	Myelitis
Chronic pain	Narcolepsy
Degenerative disorders	Osteoarthritis
Depression (clinical; reactive)	Parkinson's disease
Drug addiction (incl. Rx)	Partial paraplegic
Dystonia	Reflex sympathetic dystrophy
Epilepsy	Repetitive strain injury
Fibromyalgia	Restless leg syndrome
Graves' disease	Spasticity
Lupus	Spina bifida
Metabolic disorders	Stroke
Migraine	Structural flaws improved (e.g., scoliosis)
Motor neuron disease	Tourette syndrome
Multiple sclerosis	Vestibular disorders

The 25 years of practice using the same techniques with consistent results led Ware to conclude that the therapeutic regime stood firmly as a standardized treatment: the same approach is used with all individuals regardless of their presenting issues. The uniform methods create catalytic initial conditions that evoke the same kind of natural process that benefits each individual, but in ways the body evidently self-tailors to meet its individual needs. Observation consistently indicates that those initial conditions, once set, reliably trigger a universal response in the human nervous system (Ware, 2010a,b, 2011a,b,c, 2012; Ware et al., 2013: Soc. Chaos Psych Life Sci, Internatl Nonlinear Sci, Adv App Phys Matl Sci Congress) Since the treatment stimuli are standardized, the variables lie with and within the individuals, whose bodies can display well-known patterns of dynamic systems, introduced next.

## System transition dynamics

Until the last several years, it was not evident that clients' systems could or would regularly display well-known global patterns of dynamic systems. This was because while Ware had privately permitted his own system to “go global” in response to the therapy's initial conditions, he was hesitant about clients' potentially strong reactions to experiencing large-scale uncontrolled movements. Therefore, clients were not previously coached to relax all system restraints to the same degree now encouraged, and their systems would display much more subdued dynamics, subdued in variety and in velocity. The comparison of health benefits between previous and recent years indicates reliable results whether or not a client is coached to relax into the full process of chaotic dynamics; for example, the power-lifting champion mentioned above was not so coached. Even when more recent clients do not, despite encouragement, relax their systems that far, significant health benefits are realized (e.g., Client 3 at http://f1000.com/posters/browse/summary/1093372). Ware observes that results are stronger and achieved sooner with the global dynamics. Such clients' systems are more robust in their environment and resilient to world noise than others.

As this adjustment in coaching method yielded not only quicker health benefits but also more clearly observable patterns across clients who relaxed more globally, Ware looked to chaos and complexity theory explanations to describe what he learned were global patterns nonlinear dynamic systems could display. Our discussion of those pronounced patterns refers to client systems in which enough restraints are relaxed to display them in easily observed fashion. Until studies are designed to establish otherwise, we assume the same dynamics transpire self-similarly with more restrained clients at some scales too small for such casual observation.

Under the therapeutic circumstances described further below, the body's responses to the triggering initial conditions evidence a combination of tremulous and chaotic behaviors, with localized attractors of activity in a variety of regions, followed by observably-greater levels of system coherence. The individual is in continuous control of stopping the movements at any time, because these are neither seizures nor pathological tremors, and indeed are far more complex system behaviors than either of those. For that reason, the name formerly associated with the therapy (Ware K “tremor”) has been replaced by “health trigger process.” As adjuncts to this discussion, we urge viewing videos of some of the dynamics at http://www.youtube.com/user/generationshealthy, posted with written consent of clients (note, these may evoke reactions of cultural repulsion since most of us are conditioned to control our movements). Categories of observable dynamics are: (1) rapid, involuntary, under-skin waves of skeletal muscles' contractions and relaxations (“common” fasciculation of motor neuron activity), followed by (2) limb and/or torso tremulousness, (3) mild and/or vigorous random fluctuations in limbs or torso, (4) mild and/or vigorous stochastic chaos or periodic patterns in one or more jointed regions, and (5) abrupt changes in activity location, symmetries, velocity, and in-phase/anti-phase relations. The tremulousness of (2) is benign physiological tremor indicating interactions of various oscillatory sources in the neuromuscular system as one responds to instances of exercise, fatigue, anxiety, or certain body or limb postures (Morrison and Sosnoff, 2010). To measure and validate the specific nature of each of the progressive dynamics would require kinetic motion tracking; to date, we have relied on visual observation.

General explanations of the patterns observed above are well established, rooted in the nonlinear dynamical nature of complex systems. As inherited from Haken's theory of nonequilibrium phase transitions, the nature of the system is to remain poised on the edge of instability. This underlies the nervous system's flexibility to switch its spatiotemporal patterns on demand when interactions with the internal or external environment change (Kelso, 1995). The loss of stability triggered by an interaction enables and is marked by the appearance of new behavioral patterns, generically called emergence. Specifically, emergence is a stability transition: the new patterns arise because changes destabilized the previous patterns (Iberall and Soodak, 1998). Diverse patterns derive from diverse attractors operating within the system. Pattern changes to any affected region or system are chaotic transitions, altering its entire attractor layout as new attractors form and old ones “fall into ruin” (Kelso, 1995; Tsuda, 2009), and such chaotic itinerancy is proposed as universal dynamics in such systems as the body (Kaneko and Tsuda, 2003).

Complex living and non-living systems share many properties, and one vivid illustration of the dynamics of the general properties above is a several minute film of the gale winds-induced collapse of the Tacoma Narrows Bridge in 1940 (http://www.youtube.com/watch?v=j-zczJXSxnw). This shows transitions from mild to vigorous alternations of increasingly more of the system components as they move chaotically within their structural constraints. This is analogous to what the body does in this therapy. However, when the body's transitions enable it to escape the restraints of an unhealthy attractor, the body does not fall into ruin, as the bridge did, but moves out of chaos to coherence with a different attractor layout, much as the re-structured remains of the bridge did when the loss of structural integrity gave over to gravity's attractor and it came to rest.

Chaotic transitions to/from attractors and their behavioral patterns, rooted in collective neuronal action, are the “how” of human systems' self-organization (Thelen and Smith, 1994; Kelso, 1995). Also well described are dynamic transitions in coupled nonlinear oscillators in living as well as nonliving systems (Matthew et al., 1991; Cross and Hohenberg, 1993; Kelso, 1995), including in human limbs. Most closely related to this present discussion, limb transitions, including *within* multi-jointed limbs, display coupling, periodic temporal symmetry, in-phase and anti-phase switching and enhanced phase fluctuations, and sensitivity to degrees of freedom (Buchanan and Kelso, 1993; Kelso, 1995; Fink et al., 2000; Fuchs and Jirsa, 2000).

It has been said that there is no better evidence that self-organizing processes in the nervous system underlie coordinative changes than fluctuation enhancement, the hallmark of a nonequilibrium phase transition (Kelso, 1995). It seems the human system as a whole is “coordinating itself” during self-organizing processes in this therapy. However, due to the sheer number of the body's simultaneous processes and their multi-scaled relationships, it is commonly concluded that it is thus-far impossible to account for them all. Thus, the foregoing general descriptions of transition dynamics take us only so far in hypothesizing explanations for the nature and effects of this dynamic therapy, requiring us to turn to specific knowledge domains.

## Theoretical foundations

Our efforts to develop the hypothesized explanation are both facilitated and hampered by the state of existing knowledge across the biological fields of study. While each lends degrees of explanatory power, no existing knowledge area alone has a scope of explanatory power to explain everything we observe. Pragmatically, we must take a cross-disciplinary approach to develop our explanation. We begin with an overview of current issues in the pertinent major fields focused on recognized knowledge gaps, which, when filled, will surely develop this initial explanatory effort. We then introduce major theoretical orientations that grounded our effort to develop literature-based hypothesized explanations.

### Current issues in relevant disciplines

A current theme we glean from biological sciences concerned with the human system is one of persistent calls for higher level understandings of conceptual foundations and organizing principles to inform methods: new *ways and means* of understanding the human system. Answering these calls becomes possible, of course, because only once we stand on a recent platform of new knowledge can we identify the next unknowns.

Now that the long recognized ubiquity of fractal temporal dynamics is thoroughly assumed, the notion of fractal physiology has shifted from the status of hypothesis to an evidence-based paradigm of health (West, 2013). Central to fractal physiology's health relationship is the now widespread recognition that fractal variability means system complexity, and system complexity means a healthy biological system with normal chaotic behavior (Hong et al., 2006; West, 2013). While these are established as features of vital adaptive capacity (Lipsitz, 2008), unanswered questions include the origin and significance of those ubiquitous temporal dynamics, why their variability de-complexifies in illness, disease, and aging (Lipsitz, 2008; Seely and Macklem, 2012) and perhaps ironically, why such complexity in healthy individuals decreases with acute resistance exercise rather than increases (Heffernan et al., 2007; Izquierdo et al., 2009).

While temporal dynamics expose physiological complexity, our knowledge complexity needs its own increase to understand brain level functioning (Burggren and Monticino, 2005). Calls to understand the brain better via its multiple spatial and temporal scales do not promise to answer other calls to make connections among the micro to macro levels, connections which should unify interpretations of the meaning of those spatio-temporal scales (Andino et al., 2011). That there are connections structured in the neural system's ubiquitous functional hierarchies is presumed, but how their information processing is implemented remains unknown (Yamashita and Tani, 2008). There are calls for neuroscience to avoid physiology's trap, which was characterized as measuring increased complexity only by the number of temporal and physical structures and processes, rather than also including not only the number of transactions in sensory discriminations and behavioral alternatives (Burggren and Monticino, 2005) but also the relationships and interactions of those transactions to account for emergent behaviors (Andino et al., 2011).

Despite ubiquitous use, the concept “emergence” remains stuck in its black box (Goldstein, 2002, 2007; Burggren and Monticino, 2005), though dynamics of emergence are pivotal phenomena to understand in systems biology (Mesarovic et al., 2004) and across the disciplines. Systems biology must answer fundamental questions and integrate prior answers to do so. As in neuroscience specifically above, key questions include how macro to micro level functioning relate and how subsystem interactions coordinate (Mesarovic et al., 2004).

Though there are calls for systems biology to adopt complex systems science concepts as a way forward (Melham et al., 2013)—even while nonlinear systems analysis itself is considered still relatively primitive (West, 2013)—progress is also delayed by the reductionistim vs. holism debate that has been a plague in very many disciplines. Over 40 years ago, Koestler (1967) intended his general systems properties of open hierarchical systems of holons (holarchies) to end such arguments: his approach reconciles both sides of the debate by integrating them, as with theories of biological and scale relativity (Noble, 2012). A new biology should be integral, multilevel, and dynamic enough to integrate the vast amounts of data produced across the biological fields (Elser and Hamilton, 2007) and to agree on organizing principles is increasingly urgent (Noble, 2012).

Since it is essential to understand the nervous system's central role, there is urgency to discover the embedded order that underlies ubiquitous neural oscillations and emergence of chaotic transitions and synchronization (Steinke and Galan, 2011), and dynamics' related cognitive functions (Tsuda, 2009). Finding the basis for such unifying orientations demands a search for organizing principles to understand how systems adapt, perhaps more so than for modeling predictions (Mesarovic et al., 2004). That search should include how information moves, to determine relations among system criticality, complexity, and information transfer (West, 2013).

Information theory both seeks and may be offering organizing principles. Having established the ubiquity of information processing because nature intrinsically computes (Crutchfield et al., 2010; Wiesner, 2010) and does so logarithmically (Sun et al., 2012), it suggests many domains of research may not recognize their “compounding problems of defining and measuring information processing and dynamical systems” (Crutchfield et al., 2010, p. 1). The statistical foundations of information theory have been proposed as a unified framework to conceive how the brain integrates information in a selectionist system fashion (Tononi et al., 1998). Information-theoretic reframing may help open the black box of emergence: if discussion of the evolution of information replaces discussion of the evolution of complexity, “perhaps then we can find the general principles that have eluded us thus far” (Adami, 2012, p. 50).

Despite calls for new conceptual foundations and methods to move the biological disciplines forward, it seems many integrative insights have gone largely ignored as debates continue (Gatherer, 2010) despite urgency to agree on organizing principles. Further developments that respond to these calls will surely aid efforts to account for the human system's genius in healing its own disorders and injuries when it has the conditions to do so. In light of these current issues, we draw from across disciplines to break ground needed for explanations of the therapy's phenomena, introducing our major theoretical orientations next.

### Major theoretical orientations

The foregoing issues indicate insufficient breadth of explanatory power to meet the needs of our present effort. To hypothesize about how and why this therapy could evoke whole-system healing dynamics means starting with a high level consideration of the whole system's nature, which we do by integrating several major theoretical orientations. We draw from Koestler's (1967) holarchical systems theory for our philosophical perspective, Soodak and Iberall's (1978) homeokinetic theory for a physics perspective, and Miller's (1995; originally published 1978) seminal general living systems theory for a biological perspective. We turn to Alexander and Globus' (1996) edge-of-chaos brain dynamics as a theory of system recursivity for a neurological perspective, and from information theoretic perspectives, we construct and adopt an integrated approach to information. Consistent with the fractal physiology paradigm, we take general properties of nonlinear dynamical systems as givens, e.g., self-organization, system behaviors having sensitive dependence on their initial conditions, multiple interdependent variables influencing behavioral responses, and self-similarity. These concepts support a dynamical integral orientation toward understanding complex systems, where “a healthy state can be represented as a robust global attractor, with many parameters, and other attractors which underpin its existence and maintenance [such that] the treatment of multi-factorial disorders and diseases and the promotion of good health can be seen as nudging the disordered body processes toward good attractors” (Tasaki, 2013, p. 2).

#### Holarchical, homeokinetic, and general living systems theories

Koestler specified an open hierarchical system (OHS) comprised of holons that are simultaneously both whole systems and whole-parts of other systems. In such holarchies, the primary whole is the OHS organism under scrutiny (e.g., an atom, an organelle, a human, a society), which is a multi-level hierarchy of semi-autonomous sub-wholes connected in branching networks. It is difficult to find neurobiological descriptions of *holarchical* relationships. Where there are descriptions, integrative analyses follow (e.g., Sokolov, 1997; Bolser et al., 2006; Bonis et al., 2011; Panksepp, 2012). The critical difference between holarchical and hierarchical relations is the semi-autonomous nature of the holon, which leads to homeokinetic theory.

By pairing reductionism with constructivism, Soodak and Iberall described hierarchical levels of organization of natural phenomena via thermodynamic propositions to explain levels' relations among atomisms (holons). Their scientific scaffolding of the homeokinetic field provides conceptual linkages across a levels of organization from elementary particles below to cosmology above, i.e., from quarks and electrons to cells, brains, organisms, societies, bodies in the universe and the universe itself. Homeokinesis is homeostasis achieved via a dynamic regulation wherein the mean states of the internal variables are attained by the physical action of [thermodynamic] engines. Five thermodynamic propositions account for the expansive homeokinetic field (p. 580): ensemble mechanics implies thermodynamics, atomistic ensemble below implies continuum above, continuum below implies superatomisms above, atomisms below implies superatomism above, and continuum above implies fluctuations–generally atomistic–below. The statistical mechanics involved relate the dissipation, consistency, scales of stress, space-time scale continuums, and constraints on degrees of freedom at each level. The homeokinetic continua reinforce recognition of how dynamically and continuously interrelated the body's composition is. The general theory of living systems provides both structure and process for these relations.

**Figure 1:**
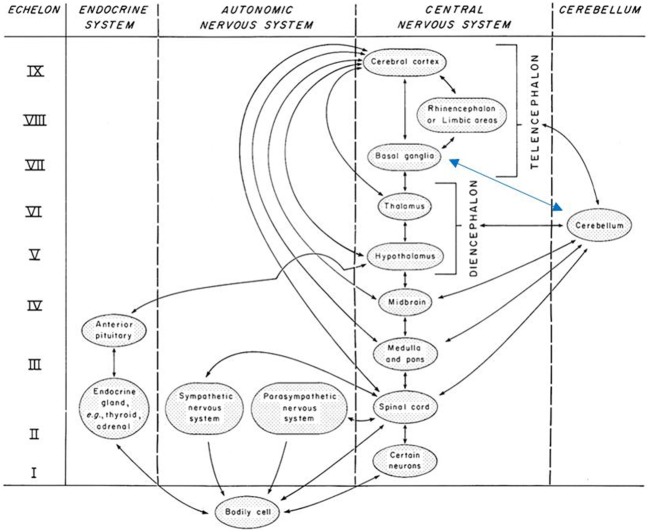
**Nine echelons of decider subsystems in higher mammals**. Note: Reproduced from original Figures 8–6 *Echelons of the decider subsystem of higher mammals*, p. 423, in Miller (1995), with written permission of copyright holder James Grier Miller Literary Estate. Adapted with blue arrow to reflect subsequent findings of reciprocal relationships of basal ganglia and cerebellum reported in Koziol et al. (2012).

The multi-level complexity of the human system depends on not just linear, but also nonlinear communications and behaviors, which in turn depend on social and natural environments for viability. Based on empirically-founded details, Miller exposes the *fractally-patterned* hierarchical structure of these relationships in systems ranging from cells, organs, and organisms to larger social scales. Clearly, holarchies are givens here. He posits universal properties across (multi-scale) systems; chief among them are 19-20 critical subsystems for processing matter, energy, and information. Some cross-scale system components that play roles in our work here include associative, memory, and decider subsystems. The number of *echelons* of decider subsystems depends on system complexity, e.g., 9 in higher mammals, 2 in the cell (Figure 1). We emphasize Miller's decider subsystems because we do not find the concept in use elsewhere but believe it is instrumental to consider in hypothesizing about the therapeutic processes.

That the detailed level of self-similarity of components, structures, functions, and processes across system scales is already identified—and is of course susceptible to updating—enables us to make certain generalizations. For example, “every internal mechanism of a physiological network is necessary to maintain either the structural or functional integrity of the organism” (West, 2010, p. 2) because “each level of the nervous system is an information link to the organization of action” (Schulkin, 2011, p. 64). Thus, if we consider interaction as a measure of complexity, nonlinear interactions between structural components that interact unequally or differently have to be considered (Burggren and Monticino, 2005). Physical interactions, by definition, depend on information, and the subsystems are taking in, processing, and transmitting information at all their concurrent (unequal and qualitatively and quantitatively different) time-and-space scales. Such complex flows of information would appear to necessitate recursions throughout the fractally hierarchical system.

#### Edge-of-chaos recursion theory

A theoretical orientation to system recursions appears underutilized in biological systems science, perhaps because the notion is subject to different interpretations (Martins, 2012) and conceptual difficulty (Wolfram, 2002). Recursive dynamics form a central premise for attempting an explanation for the therapeutic dynamics and evident effects. Alexander and Globus (1996) made an unrivaled chaos-theoretical explanation of dynamic multi-scale cascades of recursions that in our view account for the *why* and *how* of observed phenomena. Explicating recursively-organized neural dynamics, their “recursive vision” of the brain's between-scales influence is a requisite explanation for us to include. The system's recursively-embedded modularity accounts for “a universal mechanism of self-similar neural dynamics extending from individual neurons to the whole brain … a universality of dynamic which is also a structural and functional diversity” (p. 68). “The result is a *self-tuning entity which achieves this self-tuning by a multiply embedded set of processes which are continually dropping in and out of the whole and changing the way they influence the whole* (Globus, 1992)” (Alexander and Globus, 1996, p. 68, emphasis in original). This describes a system “poised at the edge-of-chaos [that] is neither too ordered and thus unchanging, nor too chaotic and so incoherent” (p. 39) where “each subsystem has to strike a balance between preserving its own local states and transmitting influence to other sub-systems” (p. 59). Smaller scale attractors correspond to diverse perceptual and fine-grained motor mechanisms, while larger scale attractors relate to the variety of cognitive, attentive and motivational states (Alexander and Globus, 1996).

Via Gregson (1992), the complexity of cascading recursions across multi-scale system components and their associated itinerant attractors becomes evident when (A) regarding the cascade as a slow long feedback loop within which fast feedforward loops are nested during which (B) transformations vary from one-to-one or one-to-many in producing (C) both ordered structures and chaotic patterns. Extending foundations built by others, Alexander and Globus explain how the multi-scale cascades of attractor changes are triggered in the global system. Its behavioral shifts go from limit cycle attractor to chaotic attractor. These shifts vary depending on the varying relationship of two conditions: (A) density of interaction within a neural system and (B) proportion of subsystems engaged in chaotic activity. As a result, this involves more than a shift in between-scales influence: the influence “is now a *projection* from ordered systems and *reception* by chaotic systems” (p. 65, emphasis in original). The recursive picture that results “is of multiple scales of a neural system, each poised near the edge of chaos. Destabilization of the dynamic at one scale of organization to a chaotic regime can lead to cascades of chaotic shifts up and down the neural level structure. This corresponds to an opening up of influence across the scales of neural systems, and a brain-wide receptive mode. Coherence emerging at one scale of organization can result in cascades of ordered shifts up and down the neural level structure. This corresponds to shutting off influence between scales of the neural system, in a brain-wide decision mode.” (p. 65).

As indicated, such cascading shifts up and down the edge-of-chaos system transpire over different time scales (Gregson, 1992) because long-term memory of the underlying processes can be quite different (West, 2010). Functional correlates in the nervous system help account for the complexity when methods account for “a partitioning of the system's parameters to encode time varying partitions of the ensemble density. The first set of system quantities that change rapidly (could correspond to neuronal activity or electromagnetic states of the brain and change with the timescale of milliseconds), the second set change more slowly (over a timescale of seconds, these could correspond to the kinetics of molecular signaling in neurons), the third set that change slowly, (like long-term changes in synaptic connections during experience-dependent plasticity or the deployment of axons that change on the neurodevelopmental timescale).” (Friston et al., 2006, p. 75).

Recursive dynamics form a central premise for attempting to explain what the system is doing during the therapy's dynamics. This premise is especially important in a systems biology perspective since the dynamics do not evidence any “privileged level or scale of causality” due to multi-scalar biological relativity (Noble, 2012). Information theory gives insight into how information is dynamically processed throughout these shifting cascades across the holonic, multi-scale stratification of complex systems, and provides our final theoretical orientation.

#### Information theory

Our orientation from information theory focuses on how the human system and its subsystems dynamically process information hierarchically, holarchically, and recursively. We discuss information and its processing, bypassing debates about distinctions between computation and information processing, which are different but often treated otherwise (Piccinini and Scarantino, 2011). This field asserts that information cannot exist in a vacuum, but has a physical substrate and therefore *is* physical (Miller, 1995; Adami et al., 2000). For example, the synthesis of information from lower-scale holons like neurons becomes neuronal network-produced synergistic information inputs to higher physical levels of the system (Adami, 2012). Indicative of a universal process, this example is like a template for understanding how both information *and* the capacity for processing it are *that which evolves* (Adami, 2012). In this case, one holon (the ensemble of neurons) determines how information from lower-level holons (neurons) is coordinated into a synthesis at a higher level, and when the information reaches the various next-relevant systems, their decider subsystem(s) make discriminations, create new information, and transmit results to the next-destination system(s) for different operations. Thus, at each moment, information is transmitted and coordinated and new information thus created, to such an extent that information is considered essential currency for organismal fitness (Adami, 2012).

How do we understand the *nature* of physical information? The literatures assume different answers. We consider it useful to separate, and then integrate, physical and psychological information (Sokolov, 1997). Thus, as others have done, we can distinguish between information and the meaning or significance assigned to it. We say that the meaning-making process includes, in Adami's terms, the capacity for processing information. The significance-determination involved in meaning making demands the capacity to do so, and results in generating further physical information. In living systems theory, information in the Shannon-Hartley sense is the degrees of freedom to choose among signals, symbols, messages, or patterns to transmit (Miller, 1995). Whether one uses implications of Miller's echelons of decider subsystems or some other premise, clearly the choice process is very different for the whole human than for the cells comprising its system—a matter of scale, structure, process, component, and function. The surface appearance of disparate common properties between and across scale, structure, process, component, and function, may be one reason some members of related disciplines mentally construct a physical-psychological divide. While nonlinear psychophysics (Gregson, 1992) never assumed such a divide, more recent work “reconciles the paradox” of bridging the perceived divide between objective and subjective information (see Friston et al., 2006; Denning and Bell, 2012; Panksepp, 2012; Sun et al., 2012).

Because Shannon ignored meaning, his theory could not account for the origin of new information (Denning and Bell, 2012), and reliance on the inheritance from Shannon seems to inhibit integrated system-information understandings. Rocchi's model (as cited in Denning and Bell, 2012) posits that information always has two parts, objective *sign* and subjective *referent.* These constructs reconcile confusions in the literature (Denning and Bell, 2012). The new association between a sign and a referent is the meaning assigned by the organism to them and is stored by the system as new information, new representations in the brain and associated subsystems. Physical interactions at each scale depend on information because “each level of the nervous system is an information link to the organization of action” (Schulkin, 2011, p. 64). Every dimension of the system is taking in, processing, and transmitting information at all their concurrent time-and-space scales. With information transmission across and between such scales, such complex flows of information would appear to necessitate recursions throughout the system. In light of these and related information-theoretic orientations, we refer to information in an integrated sense when we draw from this field in our hypothesizing.

## Therapeutic method description

In contrast to conventional physical therapies, this therapy involves no physical manipulation of the treated individual. Individuals perform the exercises on their own with instructive coaching support. The exercises are not tailored to particular presenting conditions: the same approach is used regardless of age, injury, medical history, or disorder. Treatment is a phased process. Session frequency during the brief initial treatment period may vary from daily, to alternating days, to several days apart, depending on the client. In the remainder of the first month, clients begin the self-administered, standardized progression of ongoing exercise programs for strengthening and maintaining physical and executive control.

Here, we outline the equipment, core activities, and general therapeutic premises. Initial therapy involves three models of resistance exercise equipment. No stretching or warm-up exercises are needed or permitted before using them. Early phase equipment is the leg press (which may be substituted with the seated leg curl), lateral pull down, and pectoral fly, each loaded with *very* light weight, most often the equipment's minimum. Limb movements have only two degrees of freedom (*df*) (up-down or forward-back) on the first two, 4 *df* on the pectoral. The initial assessment session(s) evidences the person's chronic system condition and how it interacts with and evaluates its environments in general. The specific activities and equipment used at the beginning of treatment are expanded after initial sessions' effects are assessed. In the next treatment phase, in addition to these and still very lightly weighted, equipment includes the seated leg curl and chest press, and then so-called “cardio” exercises (this nuisance term promoted by the exercise industry un-holistically implies the cardio-vascular system is separate, requiring its own form of exercise). In the reinforcement and maintenance phase of the program, additional equipment with prescribed repetitions and weights is introduced.

Individuals in treatment perform the following activities with trained coaching support (see a coach-trainee session at https://vimeo.com/75996645, access code *neurotricionalsciences*). (1) Learn to relax physically, mentally, and emotionally. (2) Adjust position for proper contact points and symmetrical balance on equipment. (3) Maintain upright posture. (4) Close eyes (recommended). (5) Perform *ultra-slow* movements. (6) Form and operationalize the intention to distribute effort and energy throughout the body. (7) Exert executive control to: (A) maintain ultra-slow speed and balance; (B) engage the processes of encountering resistance per coaching instructions (e.g., to pause, stop, re-start, modulate); (C) allow and relax into the varying intensities of random, chaotic, or rhythmic physical movements; (D) when instructed to do so, terminate various movements in a calm fashion. (8) Respond to instructions (e.g., adjust posture, relax, open eyes to observe and rebalance positioning, or respond in a particular way to body's movement or emotion). (9) After early treatment phases, practice control to maintain poised balance between slipping into the chaotic dynamics and not doing so.

Ultra-slow movements are performed during the initial stages of the therapy. The slow speed's role for the central nervous system is analogous to a good mechanic listening to an engine's idling state: at slow speeds, imbalances are detectible. At high speeds, rapid revolutions mask the symptoms of underlying imbalances. Daily human life tends to run at high throttle. In contrast, by moving a light load very slowly with few *df* on each piece of equipment, the nervous system can detect and become sensitive to its own initial conditions. This is prerequisite for the system to then adjust and monitor itself. Its next behaviors—during and after each therapy session—are dependent on those progressively-changing initial conditions.

Clients with slumping posture or muscle tension are coached to straighten, relax and practice executive control to correct them. If a slight dynamical movement in limbs or torso is observed during a repetition of a movement, the client is asked to suspend activity at that position to permit the dynamic response to surface and evolve. Such mild indicators are the system's first hint that a person is beginning to remove physical-emotional restraints and unbridle their system. To cease movement in unconstrained, relaxed poise sets the conditions for a full, or fuller, range of system dynamics to release. Often, just before such release, the person reports that the mild weight feels very difficult to move, usually expressing anxiety about it. It feels harder to move even light weight at ultra-slow speeds because it costs more effort and thus energy.

This overview implies the method's key therapeutic premises. The overarching premise is that certain conditions evoke transitions into and out of system-controlled chaos, requisite for the system to reorganize itself to healthier balance. The initial treatment phases are restricted to exercises that expose humans' two most vulnerable and thus most protected areas: the chest and genital regions. Use of the leg press requires spreading the legs at least to shoulder width in an upward-slanted position, with arms alongside the body. The lateral pull down and pectoral fly require feet to be positioned on the floor at roughly shoulder-width distance, and the arms to be fully extended vertically and horizontally, respectively. In addition to these commonly protected body areas, individual systems display specific, often shifting protective behaviors at various times. However, small, each is significant for the therapist to ask the client to adjust a position or retry a movement. Many of these indicate hemispheric brain imbalance. For example, the left foot may turn in protectively at the same time the right foot turns up aggressively. In such a case, two attractors associated with left-right hemispheres are in conflict and need to dissipate by calmly adjusting and maintaining bilateral symmetry (as discussed and illustrated in the trainee video at https://vimeo.com/75996645, access code *neurotricionalsciences*). Another common type of example, this time with a partial paraplegic, was the knee of the weak right leg that had been without sensation for 25 years turning inward on the leg curl for protection, and the stronger left leg's foot turning inward in a long-term “big brother” mode to protect the weaker leg. Whenever asymmetry is observed, the client is instructed to “keep your thoughts in the middle” without specific attention to the body parts (energy follows thought) to naturally manifest bilateral stability. Client goals are to practice maintaining the same emotional tone throughout all movements, controlling arousal that drives protective imbalances and building system robustness to engage the environment with composure. A final general premise is that of attending to the smallest indicators. For example, in one session several weeks into his treatment, when the same partial paraplegic was seated on the chest press, the therapist noticed the little finger of one hand was lifted in a curve off the handle during a movement. When instructed to re-perform the movement and to adjust the finger to join the hand's light position on the handle, the client's system burst into high velocity coordinated leg activity of “running” while seated (e.g., Roy et al., 2012 discussion of locomotor central pattern generators), with observable enhancement in his still-developing walking skills thereafter. Every little asymmetry means something to the system because it is generated by the system and indicates some area the system will attend to when afforded the conditions to do so.

Other premises include the following. (1) Restricting movement to very few *df* means individuals are confronted with their reactions to any feelings of exposure, frustration, or exertion. (2) Confining the pace of movement to the ultra-slow rate means the system *has time to experience* reactions physically, mentally, and emotionally. (3) Reminding individuals to relax and maintain composure means increased flow of system information because by relaxing, they inhibit their conditioning, which typically favors energy-conserving protective strategies. (4) Using new forms of executive control to replace habituated forms of executive control means chaotic responses can emerge. (5) Encouraging chaotic responses means setting conditions for reorganization and transitions out of chaos, which include learning health-sustaining forms of executive control. (6) Ensuring the therapist is trained in the purpose and premises of the methods means individuals receive individually tailored support and coaching while using a standardized therapeutic method. (7) Using a standardized method means individualized benefits are realized with consistency. In varying degrees of intensity, predictably-observable nonlinear dynamics emerge in the systems of all individuals who perform these activities under trained guidance. Our hypothesized explanations for the dynamics and their health effects begin next.

## Hypothesizing the body's genius

Our initial focal areas for hypothesizing explanations of the therapeutic phenomena are good posture, ultra-slow movements, and the associated executive functions performed by the individual. The initial conditions set by these concurrent activities destabilize and thus open the system to the phenomena in an individual who is open to improved health, as long as effort toward good posture is maintained and proper coaching is supplied. It is necessary to turn to the literatures associated with these activities to formulate and propose well-grounded explanatory hypotheses for their contributory roles in clients' health processes. To help frame this health-fostering picture, we first sketch a contrasting pathological picture to contribute to our overall effort in this major section. We then develop the hypothesized explanations for the roles of the key activities and summarize them before presenting the final two areas of hypothesizing. Those final topics concern living system interactions with inanimate structures, then possible explanations for the speed of information and healing in this therapy.

### Dysponesis

Dysponesis refers to a reversible, intermittent or continuous, physiopathologic state of errors in energy expenditures within the nervous system (Whatmore and Kholi, 1968). Selected to distinguish the systemic nature of the state, which may accompany other illnesses, the name of the hypothesis means faulty or wrong (*dys*) effort, work, or energy (*ponos*). While the term seems to have been abandoned and its early treatment evolved into forms of bio/neuro and performance feedback (Othmer, 2010, http://www.eeginfo.com/newsletter/?p=554), we emphasize how the condition was explained because we suspect it points to some of the system's self-reorganizing efforts during therapy. Thus, we recap the hypothesis to under gird explanations that follow.

Effort (ponesis) refers to producing action potentials (nerve impulses) in the voluntary nervous system. Classified efforts are performing (motor tasks), bracing (on-guard/protective response), representing (associative memory, ideation, self-signaling), and attention (impulses from some sense organs are given more influence on nervous system function than others). If misdirected, energy expenditures in those efforts are “covert errors,” functionally disturbing neurophysiologic reactions almost anywhere in the system when they form dysfunctional response patterns.

“The detrimental influence of these misdirected efforts results from the fact that action-potentials constituting effort follow not only the well-known pathways from motor and premotor cortex to anterior horn cells, and thus to muscle fibers, but they also feed signals (by way of side-branches and feedback mechanisms) into the reticular activating system, the hypothalamus, the limbic system, and the neocortex, thus producing widespread additional effects. These signals exert excitatory and inhibitory influences that are inappropriate to the immediate objectives of the organism. The result is an interference with many aspects of nervous system function including the organism's emotional reactivity, its ideation, and the regulation of various organs of the body.” (Whatmore and Kholi, 1968, p. 105).

Since the efforts composing Dysponesis “constitute one process in a complex system of interacting processes and can disturb the entire system” (Whatmore and Kholi, 1968, p. 107), healthy corrections should help heal the entire system. As essentially learned responses the system had developed to cope with its environment, such physiopathologic reactions can be corrected. Early “effort training” (Whatmore and Kholi, 1968; Whatmore and Kohli, 1974) and decades of biofeedback research indicate voluntary direction and management of energy expenditures can improve physiological conditions, perhaps because the system's efforts to “minimize free energy” (Friston, 2012) are redirected to functional pathways. Such misdirected energy in neurophysiological relationships as proposed for Dysponesis may be redirected by the system with improved functionality during therapy, and we propose this as part of our explanation.

### Posture

For the ultra-slow movements to have systemic significance, upright posture on the exercise equipment is required, thus discussed first. Postural dynamics are an information-processing system involving proprioceptive inputs (sensitivity to the body's position in space), their representations in the brain, muscle systems, and continuous feedback within the central nervous system (Kavounoudias et al., 1999). With good posture, systems move into higher energy- and information- processing states for more effective functioning. More than a biomechanical means to remain upright, the postural system is a dynamic behavioral process that facilitates other behaviors and maximizes their energy efficiency: the poorer the posture, the less efficient the information processing (Smart and Smith, 2001).

Postural asymmetry or abnormal positioning is associated with biomechanical, degenerative, and neurologic disorders (Troyanovich et al., 1998). Posture and its positive effects on respiration and oxygenation have been proposed to have powerful systemic influence in all physiologic functions, including hormone production and homeostasis and autonomic regulation (Lennon et al., 1994). Good posture is considered an essential initial condition for the therapeutic phenomena and for performing the ultra-slow movements that help trigger them. Thus, the systemic role of good posture is part of our hypothesis.

### Ultra-slow movements

An exercise movement rate of about 2.5 cm per second is ultra-slow. Just as viewing a video in slow motion enables our perceptual system to take in more information, the ultra-slow movements give the entire system more time to perceive and respond to increased levels of diverse stimuli. To perceive implies signals received and to respond means having paid attention. From the information processing standpoint, this has implications for increased complexity when attention is defined as anything that increases or decreases the firing of a neuron (Barrett and Bliss-Moreau, 2009). Movements performed at the ultra-slow rate take more physical and mental effort (effort generates more signals) and the more relaxed the state in which they are performed (open to receiving and processing signals), the more they result in more system-wide information processed for every fractional inch of movement than otherwise. Only when new information is flowing does any system change its behavior. Inhibiting *df* of motion in certain movements' efforts generates information that destabilizes the system, triggering phase transitions (Fink et al., 2000).

By contrast, information coming into the system at high rates in any domain of activity can be difficult to process and can stress the system, triggering extremes in excitatory or inhibitory dynamics depending on system-perceived significance. Regardless of ultra-slow or fast rates of information flow, interactions with the environment are modulated by the sympathetic (excitatory) and parasympathetic (inhibitory) functions of the autonomic nervous system. While inhibitory adaptability is essential to preserve a healthy range of excitatory responses even under perturbation (Maffei, 2011), and such homeostatic mechanisms operate globally throughout the system (Wenner, 2011), overload of unmanageable stimuli could foster misdirected energy expenditures of Dysponesis and the system could ignore information it needs for better functioning. Such errors can arise in biological systems that conform to the free energy principle because they selectively sample what they “expect to see” and thus behave as if they are a model of their environment because, in this way, homeostasis is supported as risks of surprise are minimized (Friston, 2012). Responses during ultra-slow movements manifest one's model of the environment and often the surprise of “model violations” (Friston, 2012) that generate disequilibriating information. We would say at such moments the system's positioning may be better characterized as in allostasis rather than homeostasis because new conditions demand change, not a return to old attractors. Behavioral dynamics discussed here play amplified roles during the ultra-slow movements, forming part of our hypothesis.

The initial experiences of ultra-slow movement against mild resistance with few degrees of freedom evidence one's chronic system conditions. The encounters reveal how individuals typically interpret and react to their (self-modeled) external environment, and representational errors can exist. Due to the logarithmic psychophysical scaling in how external stimuli are represented internally, relative rather than absolute changes in stimuli are perceived and thus only relative representational errors need correction (Sun et al., 2012). Accurate or erroneous, internal representations derive from behavioral experience from which one learns and forms memories. Given chaotic brain processes, responses to relative changes in stimuli are formed nonlinearly rather than as linear causes-effects (Freeman, 1999), thus learned responses have nonlinear associations with all representations as well. Learning, memory, and the representations they embed have dynamic, all-scales physiological origins (see Miller, 1995), and the molecular perturbations trace to neural temporal patterns because they stimulate cellular information processing (Bouteiller et al., 2011).

Thus, information flows traverse from biochemical memory and learning consolidations with numerous molecular and cellular signaling cascades, to brain regions with their own recursive cascades of system consolidations and reconsolidations (Izquierdo et al., 2006; Lee et al., 2012; McIntyre et al., 2012; Owen and Brenner, 2012; Dubnau and Chiang, 2013; Milton et al., 2013). In addition to the brain, the combined automaticity and plasticity of the spinal cord is now associated with learning, practicing, remembering, and forgetting (Roy et al., 2012). Synaptic plasticity is the mechanism of learning and memory storage (Owen and Brenner, 2012) and is essential to process massive flows of information from the stimuli of behavioral experience and assign significance to them (Dubnau and Chiang, 2013). The cascades of molecular and cellular dynamics involve complex processes of disassociating and destabilizing, re-associating and re-stabilizing (Lee et al., 2012; Milton et al., 2013), and it is well established that driving a system toward instability induces dynamic transitions in behavioral coordination (Fink et al., 2000). While experiencing the novelty of performing the ultra-slow movements under these conditions, new and increased synaptic plasticity is thus required for information processing throughout the system.

While the client makes postural and proprioceptive adjustments and responds to any associated emotional stimuli, synaptic changes of this experience-dependent plasticity (Friston et al., 2006) occur within neuronal ensembles to alter information flowing through neural circuits to correct representational errors, alter memory, and change behavioral outcomes (Dubnau and Chiang, 2013). Proprioceptive information has also been considered necessary for between-joint coordination, which is also influenced by posture (Buchanan and Kelso, 1993). We hypothesize the therapeutic context and performance of these novel ultra-slow movements create unique conditions for the system to destabilize enough to give itself system-wide attention, for the system to “take stock of its inventory” as it processes the experiences, an inventory which may include interrupted pathways, Dysponesis, injuries, or other imbalances. How individuals exercise influence during this process is covered next as we hypothesize the role of executive control of arousal and attention.

### Executive function: arousal control and attention

Performed concurrently with efforts to maintain posture and ultra-slow movements, efforts of executive function are our final focus on individual activities. During early therapeutic sessions in particular, a wide range of emotions may manifest, generating new information to process, whether from unexpected anger or frustration, fear and previously repressed trauma, stoic endurance, or surprise at discovering a sublimated worldview. Alert to and observing how client systems manifest arousal, trained Ware K therapists coach clients on relaxation because tension constrains the system. Since the early goal of the therapy is *for* the system to release the constraints of unhealthy attractor basins, therapists instruct clients on when to pause and recalibrate their response to arousal. If intense feelings of anxiety arise, the client may be told “Don't let anything happen that you don't want to happen … go back through the motion and relax the arousal, move through it slowly, it's just information, let it process on its own, you're in control.” If this appears difficult in a given session, the therapist may instruct the person to stop. This therapeutic approach expects emotions to surface and discourages dwelling on them because, as information the system has generated on its own, it has thus begun to process and consolidate it on molecular, cellular, spinal cord, and brain scales for which conscious attention is not required.

As done with clients, we emphasize the functions of arousal control and attention. Executive functions are those “an organism employs to act independently in its own best interest as a whole, at any point in time, for the purpose of survival” (Koziol et al., 2012, p. 506). The broad definition reflects recent work indicating why cortico-centric metacognitive (thinking) conceptions of executive function should be integrated with emotional-motivational (visceral) functions of subcortical areas (Taylor and Fragopanagos, 2005; Taylor, 2006; Barrett and Bliss-Moreau, 2009; Adami, 2012; Koziol et al., 2012; Panksepp, 2012). Attention and arousal are fundamental in the system dynamics of perception, representation, learning, and memory sketched above.

The activities individuals are coached to perform were enumerated earlier. All require attention, and some target visceral responses manifesting in various forms of arousal. Before individuals can pay attention to states of arousal, they must become aware of them. Individuals have stable patterns of either more or less interoceptive sensitivity to stimuli originating from within the body and interoception can intensify or contribute to emotional feelings, especially in those more sensitive (Garfinkel and Critchley, 2013). Evidence now indicates *inter*dependence of internal physiological and emotional states (Craig, 2002; Barrett and Bliss-Moreau, 2009; Terasawa et al., 2012; Garfinkel and Critchley, 2013). Emotional states have a core affect, a state of pleasure or displeasure with varying degrees of arousal, and by directing the formation and maintenance of relevant neuronal assemblies, all the brain regions and pathways involved with establishing a core affective state can indirectly constrain cortical processing, influencing which information reaches awareness (Barrett and Bliss-Moreau, 2009). Thus, physiological processes affect individuals' awareness of their emotional states, which result from their system's integration of exteroceptive information (stimuli from outside the body) with interoceptive information (Barrett and Bliss-Moreau, 2009; Terasawa et al., 2012). In continuous feedback loops, individuals' awareness of self and environment of course affects physiological processes. Studies continually increase understandings about how the complex system we call the body “knows its way around itself.”

Requiring epistemological and conceptual shifts, compelling findings (Craig, 2002, 2003) led to redefining interoception to mean the body's sense of the physiological condition of the entire body, not just of the earlier-assumed viscera. The findings have stunning implications: the lamina I spinothalamocortical system is a “homeostatic afferent pathway that conveys signals from small-diameter primary afferents that represent the physiological status of all tissues of the body,” meaning an entire neural sensory system is part of the entire homeostasis-involved network (Craig, 2002, pp. 662-663). The vertically-integrated system (e.g., see Figure 1) is comprised of autonomic, hormonal, and behavioral connections responding to all internal and external events. Pivotal network levels are spine, brainstem, and the forebrain's high-resolution encephalized (abstracted meta level) representation of the system's condition. Other perception-changing findings show the spinal cord is “not hardwired, but can interpret the combination of intrinsic activity and sensory input to adjust parameters” with plasticity and adaptability ranging from milliseconds to months (Roy et al., 2012, p. 1491). Finally, as well established in trauma research, the body retains memories of past experiences. It “keeps the psychobiological score” distributed across relevant brain regions and hormonal and endocrine systems, setting conditions for controlled arousal to invite system attention to the affects, whether already conscious or those that were originally disassociated from executive functions and sublimated to the visceral level (van der Kolk, 1996). The human system possesses thus-composed material *self-awareness*, which plays the pivotal role in our hypothesizing.

In conjunction with the body's intrinsic modalities for knowing its condition, intentionally paying attention to the body's information has effects that are part of our explanation. Since attention is the control system of the brain (Taylor, 2006), conscious attention to what the body is doing and feeling entails higher order information processing through the brain's levels and thus recursively informs all scales of the system. From our information processing standpoint, it is important to avoid conflating different levels and to understand that, and how, attention and emotion interact (Taylor and Fragopanagos, 2005; Panksepp, 2012), particularly when executive function is understood as behavioral control writ large, not merely cognitive (Koziol et al., 2012). Thus, we point briefly to three levels of executive function that have hierarchically nested and integrated roles from existential to regulatory controls (Panksepp, 2012). The primary-process level is that of basic affects, infrastructure for the other two because of its well-known instinctual subcortical memory networks upon which later learning builds. Secondary-processes are behavioral adaptations made while operating in the environment with automatic, mostly unconscious neural learning and memory mechanisms. At this level, affect states are recognized and processed by the system as signals. The tertiary-processes operate on the secondary-process information when its significance is interpreted and used in behavioral decisions. Such behaviors include movement, of course. Since it is now known that reciprocal connections exist between the basal ganglia and cerebellum, enabling a “forward model” of sensorimotor anticipation and switching mechanisms conjoined with executive functions, a more grounded notion of “embodied cognition” is conceivable (Koziol et al., 2012). Clearly, all these processes are adaptively interactive. Level 2 affects co-arise with level 1 instinctual arousals, and can be processed rapidly through level 2 learning and level 3 interpretation and thus-informed behavioral decisions and action. Automatic responses alternate with episodes of level 3 executive functions depending on the interactions with the environment (Schwartz et al., 2005; Koziol et al., 2012; Panksepp, 2012). Information processing and behavioral complexity increase when these levels integrate in action. To allow arousal to surface freely through primary and secondary processes and to control it calmly with tertiary executive functions enables persons to gradually acquire an appropriate sense of control. For example, to explicitly “have anxiety *and* know I can control it” vs. the embodied thus implicit “my anxiety *has* me *and* controls me” represents a measurably higher order of the executive function's complexity in developmental psychology (Kegan, 1982; Commons and Ross, 2008).

Given these biological structures, adaptive functions are associated with attention on immediately experienced feelings (Mehling et al., 2012) and all responses to environmental stimuli, with information processing proceeding vertically through the levels unless there are blockages (Barrett and Bliss-Moreau, 2009). The amygdala's dopaminergic “boost” to tertiary executive functions is a known factor in rapidly orienting attentional resources to novel events (Fried et al., 2001; Kaplan and Oudeyer, 2007) and is associated with reaching those functional levels (Panksepp, 2012). We consider attention exercised per instructions during the therapy is a crucial mechanism to unblock the system to process all the information generated during the exercises. Such novel changes in type and array of stimuli forces perceptive functions to adopt more complex, controlled responses. These engender more psychological complexity, which has higher information processing costs (Aksentijevic and Gibson, 2012). Attention demands of tasks affect neuronal oscillation (Sosnoff et al., 2005) as they amplify neural activity toward the attended stimulus while they reduce neural activity representing distractor stimuli, with influential biases from even fleeting, unnoticed emotion (Taylor, 2006). To manage psychological and physiological processing costs, the system needs to reduce its noise, escape attractors and migrate to new ones (Kaneko and Tsuda, 2003; Tsuda, 2009), and adjust its degrees of freedom to minimize free energy (Friston, 2012) as it updates its overall condition-awareness from moment to moment. The therapy requires individuals to replace habitual goals by learning and practicing more complex “goal modules” (Taylor, 2006) involving physical and executive behaviors that inherently demand the coordination of more information. Indeed, as the information to be processed evolves, the entire system is performing more, and more complex, tasks at each of its multiple scales (Adami, 2012).

Thus, we propose the breadth of executive functions over arousal and attention during well-maintained posture and properly performed ultra-slow mild resistance exercises dynamically amplifies and complexifies system information. Before therapeutic benefits emerge, the system has numerous multi-scale transitions to traverse. Those transitions are facilitated by executive commands to relax and “show no concern for what is happening below the neck.” As demonstrated previously, “it is only when these degrees of freedom are exposed during development by the dissolution of old, rigid forms that the organism is able to explore new, more functional solutions” (Thelen and Smith, 1994, p. 77). With constraints loosened, the edge-of-chaos system takes the necessary degrees of freedom to process its cascading recursions of relevant information in whatever way it identifies necessary to explore and implement solutions necessary in its self-(re)organization.

After early treatment phases, clients are reminded to practice executive control to maintain the poised balance between slipping into the transition dynamics and not doing so. As a physiological goal with therapeutic benefits, these efforts help individuals develop a confident sense of control over their system and its interactions with the environment. Just as the system maintains the balance between homeostasis and chaos by holding its poise on the edge of instability, so also the individual can consciously participate in that healthy balancing act toward developing and behaving out of a healthy model of self and the environment.

### Interim summary

We pause to iterate and draw together the explanations hypothesized in the work presented above. Five explanations represent testable hypotheses. (1) Coaches who have themselves participated in the therapeutic program and been specifically trained in the method and its premises are required for clients to realize therapeutic results. This can be tested with coaches who do not meet these conditions but who have been instructed to coach clients to maintain good posture, ultra-slow movement, and executive control. (2) Since a system's misdirected efforts cause a pathological neurophysiology, correctly-directed system efforts may restore healthier neurophysiology. Controlled studies targeting specific conditions such as those in Table 1, could test if the therapy represents correctly-directed efforts that ameliorate them. (3) Good posture is an essential initial condition for the therapeutic phenomena and for performing the ultra-slow movements that help trigger them. This can be tested in studies where clients are coached to maintain ultra-slow movements and executive control but not good posture. (4) Diverse, and diversely related, behavioral dynamics that accompany ultra-slow mild resistance exercises performed with few degrees of freedom play amplified roles in information processing, enabling new system inventory-taking of its conditions and triggering destabilization and transition dynamics that modify that inventory. This can be tested in controlled studies that use mild resistance exercises on the same equipment with the same weight but at conventional speeds of movement. (5) Executive control over arousal, along with effortful attention during the therapeutic exercises, is essential to release system constraints, and amplify and complexify system information at the same time. This can be tested in controlled studies where good posture and ultra-slow movements are maintained but coaching for executive control and attention is omitted.

The five method-based hypotheses, even if not disconfirmed by testing, do not explain the *how or why.* We hypothesized (Section Ultra-slow movements) that the therapeutic context and performance of the novel ultra-slow movements create unique conditions for the system to destabilize enough to give itself system-wide attention, for the system to “take stock of its inventory” as it processes the experiences, an inventory which may include interrupted pathways, Dysponesis, injuries, or other imbalances. This is consistent with the pivotal explanation given earlier, the empirically-based deductive conclusion that, by its entire physiological nature, the human system possesses material self-awareness. That conclusion is summarized as follows. Through the lamina I spinothalamocortical system it knows the physiological status of all the bodily tissues. Through the spinal cord, it interprets the total ensemble of intrinsic activity and sensory input and accordingly makes near and long term adjustments with adaptive plasticity. Through its memory-related components and subsystems, the system knows its psychobiological status through its distribution across brain regions and hormonal-endocrine systems. It would appear that client systems use their therapeutic dynamics both to “know more” about their physiological statuses and to inform a multitude of actions to improve or correct them.

The *how* is not yet clear at this interim point, thus further explanations are hypothesized next. We selected the remaining two focal areas based on how pivotal their roles seem for understanding the body's use of the therapeutic experiences and their resulting benefits. The first focuses on the interaction of animate-inanimate systems. The second explores issues around the information speed in the dynamics and healing.

### The living interacting with inanimate structures

A complementary conjunction of inanimate structures with the living system plays a central role in clients' learning how to release their habitual system restraints. The interactions are comprised of (1) the resistance exercise equipment's imposed degrees of freedom during ultra-slow movements, (2) the field of the therapist-client interactions concurrent with client-equipment interactions, (3) the client system's internally chosen degrees of external freedom, and (4) the vast range of the body's externally enacted degrees of behavioral freedom during transitions, even within—and despite, if not because of—the equipment-imposed confines.

Both the stationary and movable structural elements of the equipment can be used by the system to facilitate processes of leaving and forming attractors, especially but not only, of course, when high velocity periods ensue. These often include repeated banging of body regions against a seat, backrest, or shoulder pad, for example, before transitioning to another attractor. The system evidently needs to meet resistance *somewhere* to destabilize enough to begin to undertake reorganizational processes; true in psychological processes, it may be equally true in physiological ones. As therapy progresses, however, the form of resistance need not be confined to the exercise equipment. Once clients have learned how to release their constraints and allow their bodies to go into the dynamics, it can be done at will without the equipment. For example, clients learn how to lift low-weight free weights with arms raised above their heads and allow their standing bodies to go into the dynamics. Some can merely sit or stand normally. One very seasoned practitioner can lie on the floor, which provides structural resistance, and allow her system many degrees of freedom to go into dynamics that involve the same kinds of in-phase/anti-phase symmetry changes with limbs and banging certain regions on the floor as may happen on exercise equipment (see http://vimeo.com/user20254671/review/77365649/bad8e5f1b6).

Two conclusions follow from this discussion. The first is that the trained coaching support is required during the initial phase of therapy for client orientation and skill-building, but coach-client interactions are not required thereafter. The second is that inanimate forms of structural resistance play useful roles while one learns requisite skills. Thereafter, the edge-of-chaos system has more degrees of freedom in how it may initiate its own dynamics with or without external structures to push against. Thus, we hypothesize that the roles of therapist and equipment are facilitative in triggering the dynamics but not causative. That is, they facilitate the therapeutic initial conditions in a treatment session for the body to respond to experiences and destabilize enough to start generating and processing more information. By its nature indicated above, the body distributes and updates its information continuously to self-reorganize its physiological statuses.

### Information speed and healing

In light of the foregoing discussion and in the spirit of fractal physiology, in this section we follow the claim that behavior is a source of insight into principles of self-organization (Kelso, 1995). As cited earlier, an accepted premise is that systems' transition behavioral patterns are rooted in collective neuronal action and are the “how” of human systems' self-organization, and that such complex flows of information would appear to necessitate recursions throughout the fractally hierarchical system. In addition to our desire to understand *why* clients consistently demonstrate within the initial days of therapy early improvements in, or in some cases, full resolution of their troubling conditions, we have been curious about the causes underlying the high velocity of some of the dynamics when individuals relax their internal constraints. The central and peripheral nervous systems' functions and their extreme plasticity are common denominators here. Thus, we hypothesize possible connections between the visible velocity of external movements and the unobservable internal rapidity of the body's healing mechanisms. With the premises of recursion theory presumed, two related approaches to explain the therapeutic information's speed and healing impacts are hypothesized next: complexity matching and speed of neural information.

#### Complexity matching

Since stimulus-response mechanisms are universals in living systems, exploring the relationship of the observable system dynamics to internal healing mechanisms requires an attempt to identify stimuli, a difficult task. The individual in therapy initially moves arms or legs at a very slow rate on the equipment against mild resistance. Self-organized systems' events are usually generated by a slow motion (Allegrini et al., 2006). When the slow-moving system's response to the mild resistance becomes stressful (i.e., the light weight always begins to feel unbearably heavy) an initial perturbation event occurs. Perturbations, if meaningful to the system, destabilize it. The fast-moving fasciculation responses in surface muscle groups begin, followed by fast-rate tremulousness in the limbs. From the slow-moving initial perturbation, mild but rapid system responses ensue, associated with the original perturbation but not reducible to it. As they extend throughout more of the system, are they responses to the stimulus of the earlier perturbation, new stimuli received into deeper regions of the system, or both? They appear to be both, especially given the multi-scaled holarchical system already established as continuously processing information over different time spans.

With the focus here on dynamics' speed, we confine our scope to the rapid dynamics as stimuli into deeper regions of the system, where complexity matching can be considered. Tononi et al's (1996, 1998) matching complexity (*C*_*M*_) refers to changes in neural complexity after environmental signals are received. Once a collection of intrinsic correlations was selected to adaptively match the statistical structure of that sensory input (including that of other neurons), the repertoire critically influences how the brain categorizes individual stimuli. The brain incorporates its intrinsic contexts in its signal processing via the reentry of information flowing in ongoing recursive signaling among many sets of neuronal groups, triggering a large increase in the mutual information in many of its regions to amplify and “go beyond the information given.” Thus, the same stimulus can convey radically different amounts of information when its associations are meaningfully rich to the system via high matching complexity.

The statistically measured extent of meaning in matching complexity generalizes from the rate matching condition (Lukovic et al., 2008). When the rate matching of perturbation and system are equal, an abrupt transition ensues. For example, when there are breakdowns in the collective signal structures (e.g., model violations in Friston's terms), memory is reset through a process characterized by the time between events, the complexity matching effect (Allegrini et al., 2006). In rate matching, either (1) the perturbation event rate is larger than the system event rate and perturbations are attractors of the system events or (2) the opposite condition where perturbations events become repellors of system events when the system rate is greater (Lukovic et al., 2008). The limitations of the system as information channel determines the maximum range because the system responds based on its finite information capacity.

While highly abstract, the findings from matching complexity, the complexity matching effect, and rate matching consistently establish direct relationships between the complexity of stimuli and the complexity of system responses to matched stimuli. On that basis, we hypothesize that there is a direct correlation between the speed of observable system dynamics during therapy and the healing responses that go on within the body. Given the recursive nature of the system, we assume the observable dynamics have dual roles as ongoing stimuli and responses throughout all scales of the system.

#### Speed of neural information

The selectionist approach of Tononi et al to matching complexity, that explains how the brain operates on more than the information given, is directly related to the speed of neural information flows, which have direct roles in system healing and the rate thereof. The *how* question about healing speed remains, and we turn to other work connected with the speed of neural information.

Using a neural network model with multi-timescale dynamics, synthetic neuro-robotics experiments revealed fast and slow action dynamics with a set of primitive action patterns embedded in the fast, and pseudo-stochastic dynamics of spontaneous pattern sequencing in the slow (Namikawa et al., 2011). The faster-developed primitive patterns were coordinated during the (understandably) slower act of sequencing them. The self-organization of this functional hierarchy ensured robust action generated in the noisy environment provided, correlating with known brain action functions. As discussed earlier, encephalized topographical maps are known to have “staggering” plasticity (Kelso, 1995) to remap themselves and reorganize the temporally-correlated activity among neurons. Also as discussed, it is proposed that temporal scales differ within partitions of neuronal ensemble densities (Friston et al., 2006). The kinds of mean field models that produced those findings have been criticized for lacking biological fidelity when they fail to address non-local, distributed neural activity via the long range axonal fibers (Bojak and Liley, 2010). Individual axons have been shown to have different conduction velocities and conduction time delays are broadly distributed such that neuronal populations are connected by axonal fibers with broad ranges of velocity (Bojak and Liley, 2010). The discovery of the previously-inconceivable far reach of elongated axons accounted for the surprise of finding neural network correlations between far-removed sites (West, 2013). For example, it is known that *de novo* spinal circuits form with propriospinal neurons above and below spinal cord injury lesions to restore communications (Flynn et al., 2011). Given their vital connectivity functions, axonal growth, myelination, re-myelination, fasciculation, pathfinding, and repair are widely studied. Axonal regeneration and extension and nerve regeneration are among the requisites for nervous system repair, and combinations of drugs, stem cells, and other therapies are widely assumed required (Xu et al., 2011; Allodi et al., 2012; Konofaos and Terzis, 2013).

The speed of connections and of regaining connections quickly are key factors not necessarily achieved with such interventions. Our initial looks into the speed of growth, and silent presence, of central nervous system synaptic connections indicated three relevant phenomena. Due to the rapid recruitment and transit of NMDAR receptor transport packets (about 4 μm/min) to nascent synapses, glutamatergic synapses can form rapidly (Washbourne et al., 2002), with assembly occurring within 1-2 h (Friedman et al., 2000). Likewise, silent synapses can activate rapidly, with strong cases made for them becoming active with appropriate stimulation, although they are less pervasive in the mature central nervous system than in the developing one (Atwood and Wojtowicz, 1999). The importance of their recruitment for mature nervous system plasticity was undetermined at the time of that review and we have not found a more recent treatment of the topic.

Unsatisfied that the foregoing mechanisms were likely to generate adequate explanations, our final investigation into the question of “why so fast?” turned to non-synaptic communications via ephaptic coupling. Since chemical and electric neural communications occur with the bodily environment, not isolated in a non-organic vacuum, electric action potentials exist at both synaptic locations and within extracellular space, which also makes it difficult to distinguish which effects derive from which types of communications (Anastassiou et al., 2011). An array of methods have been used to study or model how these non-contact dependent signals propagate, variously focused on cardiac cells (Copene and Keener, 2008; Lin and Keener, 2010), cortical neurons (Anastassiou et al., 2011), the mammalian olfactory system (Bokil et al., 2001), unmyelinated axons (Bokil et al., 2001) and myelinated nerve fibers (Binczak et al., 2001). Ephaptic coupling has also been among mechanisms covered in a review of physiological and epileptic generation of high frequency oscillations (Jefferys et al., 2012) and in a study of transitions to epileptic seizure (Zhang et al., 2011). It is beyond our scope and expertise to elaborate on specific conditions and contexts addressed in those studies, but a common denominator appears to be the relationship between ephaptic coupling and increased velocity, and “reach” of signal transmission. While this area of specialized study is still developing with much yet unknown (Jefferys et al., 2012), indications are that ephaptic coupling may play a significant role in developing answers to the “why so fast?” questions associated with this therapeutic method. This seems an especially appropriate stance, given that clinical applications have long been successful using extracellular electrical neural stimulation of the central nervous system to treat a quite diverse array of conditions (Joucla and Yvert, 2012) similar to those presenting in this therapy's clients over the last 25 years.

This limited investigation into speed-related issues by non-specialists may indicate we have gaps in our understandings and assumptions. At the same time, the extreme rapidity with which new neural connection mechanisms appear to transpire in this therapy may support our proposal. We hypothesize the possibilities that (1) the “staggering” rapidity of central and peripheral neural dynamics typically referred to may be even faster in this therapy, and drive the high velocity movements of the body, and (2) that the therapeutic dynamics in general, along with the high velocity movements when they occur, create the necessary and in many cases evidently sufficient conditions for the nervous system to conduct both unusually rapid repairs and amplified communications for the body to resolve or improve a diversity of conditions within short time periods. We would not call this either circular reasoning or circular causality, because of the thoroughly recursive nature of the self-aware system.

## Conclusion

It seems to us that the overall explanation for the nature and effects of the therapy lies in nature of a complex living system, the recursive nature of the body's edge-of-chaos dynamics, the means of conducting those multi-scale recursions, and how it uses the information and energy therein. Evidence indicates and we have argued for the material self-awareness of the entire physiological system. Evolution does not select for unneeded functions and mechanisms, and many physiological regulatory systems have present or future capability for complex actions (Burggren and Monticino, 2005). Perhaps because the onset of disease and disorders is typically slow, we are conditioned to assume many of the body's healing processes are not only slow, but perhaps also not possible via natural processes. The long history of the Ware K therapy and the research to under gird the various levels of hypothesizing here lead us to propose that the self-organizing body system has the edge-of-chaos genius to enact whatever dynamic behaviors are stimulated to emerge in response to initial conditions from moment to moment and to benefit from their effects.

Operating with higher levels of system complexity has been long associated with greater health and reduced effects of aging, now a firm premise (Lipsitz and Goldberger, 1992; West, 2013). The purpose of the self-awareness would seem to be to perform continuously recursive actions at whatever complexity and to whatever extent is required to preserve and maintain the organism and proper functioning. In such recursive communication feedback dynamics with synaptic and ephaptic mechanisms, isolation of components decreases, thus preventing the loss of nonlinear feedback loops known to be caused by such isolation (Hong et al., 2006).

Such recursions may be the “engine” of the system's self-organization (and one of the keys to the persistent black box of emergence). Recursions have tripartite roles in the system dynamics: they are simultaneously means, causes, and effects of the multi-scaled cascades of information, assignments of significance, and actions throughout the human body. It seems the human system is such an expert on itself, on its own history and conditions, that self-reorganizing processes are performed recursively by the whole system *on itself*, to adjust, rebalance, and heal itself when the right set of initial conditions exist.

It may be the case that only such interventions as physical manipulation, surgery, and medication should be reliably expected to fix or heal imbalances, injuries, diseases, and disorders of the human body. Yet, the 25 years of clinical practice with this method suggest there is much yet to understand about the body's genius to fix or heal itself, too. Via such phenomena as described herein, the body itself may be teaching us new ways and means of understanding it, if we can imagine it possible for that to be the case.

“If we fail to acknowledge the potential complexity of a poorly understood system, we then mistakenly view all physiological observations as reflecting the maximum possible complexity of that system, not acknowledging the potential complexity of the system leads us to underestimate the complexity of its ultimate emergent behaviors … While the complexity of things well understood seems obvious, how much potential complexity remains undiscovered until we make observations under new configurations of physiologically relevant conditions?” (Burggren and Monticino, 2005, p. 3226).

Burggren and Monticino imply the need to entertain new levels of curiosity and expansive worldviews. These may be important to help make sense of phenomena and consistently positive results using a standardized method to treat diverse conditions, such as we have hypothesized about here. Such a worldview may entertain the possibility that it is in the very nature of the human body to possess both self-awareness and capacity sufficient to self-*re*organize its healing from even serious conditions when it has the initial conditions in place to do so. We fervently hope these hypothetical explanations motivate research programs to test these and other hypotheses, with the aim to inform basic science and clinical practice at the same time as they exploit the therapy's potential to improve human health and well-being more widely.

## Author contributions

Sara N. Ross investigated the literatures, formulated the theoretical approaches, developed the hypothesized explanations, and wrote the article. Ken Ware, as originator of the therapy, supplied the historical background and therapeutic expertise, and from his years of study, the rationales behind the methodological and dynamical focal areas hypothesized.

### Conflict of interest statement

Ross declares that the research was conducted in the absence of any commercial or financial interest. She was an independent researcher contracted by Neurotricional Sciences, Pty. Ltd. to conduct the research to produce this article. She was adjunct faculty at Antioch University Midwest during this project; the university subsequently hired her as full time faculty. Ware is the director and a shareholder of Neurotricional Sciences Pty. Ltd. which delivers the therapy he developed and trains therapists.
